# Knowledge management strategy for advancing the national health agenda in Dominica

**DOI:** 10.26633/RPSP.2017.3

**Published:** 2017-02-08

**Authors:** David Johnson, Marcelo D'Agostino, Myrna Marti, Federico G de Cosio

**Affiliations:** 1 Ministry of Health and Environment Ministry of Health and Environment Roseau Dominica Ministry of Health and Environment, Roseau, Dominica.; 2 Health Information and Analysis Unit Department of Communicable Diseases and Health Analysis, Pan American Health Organization (PAHO) / Regional Office of the World Health Organization (WHO) Washington, DC United States of America Health Information and Analysis Unit, Department of Communicable Diseases and Health Analysis, Pan American Health Organization (PAHO) / Regional Office of the World Health Organization (WHO), Washington, DC, United States of America.; 3 Office of Knowledge Management Bioethics, and Research, PAHO Buenos Aires Argentina Office of Knowledge Management, Bioethics, and Research, PAHO, Buenos Aires, Argentina.; 4 Central Statistical Office Reports on Population Census Roseau Dominica Central Statistical Office, Reports on Population Census (Roseau, Dominica).

**Keywords:** Knowledge management, information systems, public health, health systems, Dominica

## Abstract

The Ministry of Health and Environment (MoHE) of the Commonwealth of Dominica identified the need for a knowledge management strategy to advance the country’s national health agenda. The Pan American Health Organization and the MoHE conducted a rapid situation analysis of knowledge management in July 2015. The findings, analysis, and recommendations were developed jointly with a core team of the MoHE within the context of the strategic plan for health, “Investing in Health – Building a Safer Future.” The situation analysis described the overall status of the understanding and implementation of information and knowledge management activities, projects, products, and practices. The analysis also aimed to identify what critical knowledge is needed to support overall organizational goals and individual and team activities. The MoHE expects patient outcomes and quality of care to improve as a result of having a knowledge management strategy that boosts the Ministry’s efficiency and productivity.

## RESUMEN

El Ministerio de Salud y Medioambiente del Commonwealth de Dominica determinó la necesidad de definir una estrategia de gestión del conocimiento para impulsar el programa nacional de salud del país. En julio del 2015, la Organización Estrategia de gestión del conocimiento para impulsar el programa nacional de salud de Dominica Panamericana de la Salud y el Ministerio de Salud y Medioambiente llevaron adelante un análisis rápido de la situación de la gestión del conocimiento. Los resultados, el análisis y las recomendaciones fueron elaborados conjuntamente con un equipo básico del Ministerio dentro del contexto del plan estratégico de salud Investing in Health: Building a Safer Future [invertir en la salud para forjar un porvenir más seguro]. En el análisis de la situación se describe el estado general del conocimiento y el desarrollo de actividades, la ejecución de proyectos, la elaboración de productos y la aplicación de prácticas de gestión de la información y el conocimiento. Dicho análisis además tuvo como objetivo definir el conocimiento fundamental que se necesita para apoyar la consecución de todas las metas institucionales y la realización de las actividades tanto individuales como grupales. El Ministerio de Salud y Medioambiente espera que mejoren los resultados de los pacientes y la calidad de la atención al contar una estrategia de gestión del conocimiento que le permita impulsar su eficiencia y productividad. Ministerio de Salud y Medioambiente del Commonwealth de Dominica determinó la necesidad de definir una estrategia de gestión del conocimiento para impulsar el programa nacional de salud del país. En julio del 2015, la Organización Panamericana de la Salud y el Ministerio de Salud y Medioambiente llevaron adelante un análisis rápido de la situación de la gestión del conocimiento. Los resultados, el análisis y las recomendaciones fueron elaborados conjuntamente con un equipo básico del Ministerio dentro del contexto del plan estratégico de salud Investing in Health: Building a Safer Future [invertir en la salud para forjar un porvenir más seguro]. En el análisis de la situación se describe el estado general del conocimiento y el desarrollo de actividades, la ejecución de proyectos, la elaboración de productos y la aplicación de prácticas de gestión de la información y el conocimiento. Dicho análisis además tuvo como objetivo definir el conocimiento fundamental que se necesita para apoyar la consecución de todas las metas institucionales y la realización de las actividades tanto individuales como grupales. El Ministerio de Salud y Medioambiente espera que mejoren los resultados de los pacientes y la calidad de la atención al contar una estrategia de gestión del conocimiento que le permita impulsar su eficiencia y productividad.

Dominica, the largest and most northern of the Windward Islands, is only 29 miles long by 16 miles wide and has 91 miles of coastline (1). The Government of Dominica has a Westminster-style parliamentary government. The executive branch includes the president, who is elected by parliament for a 5-year term, and the prime minister, who is appointed by the president. Dominica’s legal system is based on English common law, with three magistrate courts that may appeal to the Eastern Caribbean Court of Appeal, and ultimately, to the Privy Council of London (2). The local government consists of councils with the majority of representatives elected by universal suffrage. Supported by property taxation and government grants, the councils are responsible for the regulation of markets and sanitation and the maintenance of secondary roads and other municipal amenities. The island is also divided into 10 parishes whose governance is unrelated to the local governments. The Carib territory has its own ruling council with greater autonomy.

According to the Central Statistical Office (Roseau, Dominica) the country’s population grew from 70 121 in 1991 to 70 340 in 2011,4 virtually no change in 10 years. During that same period, the natural increase (the excess of births over deaths) was 9 300. The population is predominantly (almost 80%) of African descent. Just over 4% are Carib, the only concentration of indigenous people in the Antilles.

## Health care system

In an effort to provide universal coverage and access, health care is offered free of charge at the primary level. Overseas organizations, including faith-based organizations, often bring in teams of health workers to provide specific services – not necessarily in the areas of greatest need. Health services were devolved as part of health sector reform and the implementation of Primary Health Care (PHC).

Dominica is divided into seven health districts for PHC delivery. Each district has a Type III health center and at least four Type I clinics. Each district has its own budget, but all the budgets are centrally managed due to a lack of human resources. The seven districts are grouped into two regions, each of which is supervised by a regional manager who reports into the national PHC Director.

## Health information system

A comprehensive and coordinated health information system is recognized as one of the essential steps for building stronger health systems. Information is imperative for health administrators and clinicians, especially since they strive to make decisions that will maximize limited resources and provide the best quality of care to individuals and the wider community. The many and varied needs for information, along with the introduction of new technologies, make the scaling-up process more complex and challenging for the local environment (3).

The PAHO “Regional Strategy and Plan of Action on Knowledge Management and Communications,” (4) underscores knowledge management (KM) as an indispensable tool for supporting decision-making, as well as promoting individual, social, and political changes that improve health. KM is also seen as a dynamic process characterized by different variables consisting of access to information, production of knowledge, dissemination, and training (4). WHO uses the term “knowledge management” to describe the use of technology in order to allow people to create, collect, store, recover, use, and share knowledge. The Ministry of Health and Environment (MoHE) of Dominica expects improved patient outcomes and quality of care to result from having a knowledge management strategy that produces greater efficiency and productivity and is a shared responsibility of all health workers.

The main objective of this analysis and report was to define the vision, main goals, lines of action, and priorities for the development and implementation of a Knowledge Management Strategy for advancing Dominica’s national health agenda.

## Rapid situation analysis for strategy design

To develop a KM strategy for advancing the country’s national health agenda, PAHO and a core team from the MoHE conducted a KM rapid situation analysis on 13 – 14 July 2015. Table 1 describes the various roles of the stakeholders. The findings, analysis, and recommendations were developed jointly with PAHO and within the context of Dominica’s national health strategy (3). The team discussed main goals, lines of action, expectations, and priorities for the development and implementation of the KM strategy. This report outlines the PAHO experience designing the KM strategy in Dominica.

In preparation for the 2-day rapid analysis meeting, a multidisciplinary group of experts on public health, health analysis, knowledge management, information technologies, statistics, and library services from the MoHE and PAHO was convened in March 2015. PAHO presented a questionnaire of 26 items that covered the following areas: determinants of health, human resources, healthy community approach, legal framework, information and communication technologies (ICT), health information systems, knowledge management, and MoHE organizational structure and expectations. Through virtual sessions, the questionnaire was validated by the multidisciplinary group. About 20 of Dominica’s team completed the questionnaire. The PAHO team reviewed the answers and prepared a summary of findings. The multidisciplinary group then met to discuss the findings, and to reach a consensus on recommendations for the MoHE officials.

## Summary of findings

Findings were determined in conjunction with a technical team led by the country’s Chief Medical Officer. Each of the components presented here was widely discussed in the context of Dominica’s national health agenda.

**Table 1.** Roles within the Ministry of Health and Environment of Dominica in addressing disparities in social determinants, 2015

Leader	Addressing the health and long-term care needs of certain population groups and as an employer.
Influencer	Finding win-win situations that convince other sectors to develop public policies and assign public resources that improve social determinants.
Communicator and knowledge broker	Communicating with the public and decision-makers about the impact of policies in the sectors where health, wellbeing, and productivity are involved. Building and sharing an understanding of mechanisms for reducing the social determinants of health

## Expectations

Senior management at the MoHE considered it important to transition to a knowledge-based organization, fully functional within the “Information Society,” and to reinforce the use of ICT tools to improve overall productivity and health care delivery. While the results of the analysis showed that the concept of knowledge management was not part of the organizational culture, senior management was confident that the staff, as a whole, understood and supports the benefits KM. There is also a high level of expectation that KM will facilitate diplomatic dialogue, technical conversations, and the sharing of critical information among the key players in the health system at the national and international levels.

## Social determinants of health

As stated in the national health agenda, specifically “Investing in Health: Building a Safer Future” (3), the MoHE of Dominica is aware that the social determinants of health directly impact individual health as well as social care issues. The Ministry has identified the following determinants as particularly influential in Dominica: Unemployment, Income and Social Status, Social Support Networks, Education and Literacy, Social Environments, Poverty, Relative Deprivation and Social Exclusion, Personal Health Care Practices and Coping Skills, Physical Environment, Housing, Health and Social Care, Food Insecurity, Culture, and Gender (3). All of these key determinants have implications for policies and programs concerning the population’s health. With this perspective, the health sector in Dominica identified three roles (A – C) in addressing disparities in social determinants.

### *A. Human resources*.

There is a lack of human resources in several areas due to migration of health care professionals. The MoHE identified economic and educational reasons as the main causes for migration. The MoHE currently manages infrastructure and resources with a small team of qualified people, and provides staff training through collaborating agencies and other entities. Health provider skill-sets and qualifications need to be aligned with the demands of services associated with industry growth, increases in cases of non-communicable diseases, generic population and immigration, and disaster management and response.

Training opportunities must consider a KM strategy that provides health workers with the ability to search, manage, use, and share scientific and technical information, and the availability of these tools to facilitate daily work. Although the MoHE does not have a digital literacy program, it provides informal training-related knowledge management practices. Workers have access to orientation sessions on knowledge and experience transfer as part of succession planning. There is currently a coaching and mentoring program implemented in the nursing department; its outcomes are being evaluated. The minimum skill-set for a “qualified” health worker in the Information Society include aspects of ICT tools, scientific writing, content production and dissemination, public information and social networks, and online information searching.

Regarding the migration of the health care professionals, the MoHE should have a strategy for retention, providing access to up-to-date technical and scientific information, permanent education, and knowledge transfer for retirees.

### *B. Healthy community approach*.

The Healthy Community Program deserves special attention since the KM strategy could likely strengthen its core components: partnership building, capacity building of officers and community leaders, community assessments, community profile documentation, and community participation in planning health solutions. Through the leaders involved, this initiative mobilizes communities and resources around activities focused on wellbeing and healthy living.

### *C. Legal framework*.

The existing legal framework is being reviewed. A need has been identified for more user- friendly

and supportive health legislation to assist the sector in achieving its goals. This is an opportunity to incorporate KM concepts into revised laws, for example: open access to publications, open source for ICT development, patient information protection, use of evidence and technical information in policy development and decision-making processes, adoption of open government data policies, use of electronic signature, and improving the security of health records.

Dominica has a law aimed at promoting competition in telecommunications, but it does not have regulations that directly impact on ICT in health, standards, interoperability, infrastructure, development, and accessibility. There is also legislation stating that patient records should be kept for 7 years, and that, within telecommunications, any subscriber or end-user data is confidential and cannot be used without the express consent of the owner.

## Information and communication technologies

Innovative ICT should be considered key to developing the national health plan. This document (3) was created to support the Health Information System. ICT is considered the new standard method in communications, information and knowledge sharing, training, information management, and so on, and is consistent with Dominica’s e-Government for Regional Integration Projects.

## Information systems for health

The MoHE decided to produce an integrated information system within the ministry. The Health Information Unit under the National Epidemiologist at the MoHE is charged with implementing this “Integrated Health Information System.” The initiative is based on a clear vision that access to quality information and open data is vital to providing proven and effective interventions to health workers in all health matters.

The MoHE is also in the process of revamping its Internet site for better knowledge management, taking it beyond a mere dissemination tool and repository of documents. There is also a need to develop and implement a specific social networking strategy. The MoHE considers social media to be an important tool for technical discussions and for participating in discourse as the country’s authoritative source of public health information. Therefore, developing thematic networks is critical to making it possible to know users’ opinions and interests and to facilitate evidence-based decisions for policy making in Dominica. The high level of Internet users in the country should be noted: in 2014, the penetration rate was almost 63%, with 35 000 Facebook subscribers by November 2015 (5).

## Knowledge management goals

After reviewing the findings of the rapid situation analysis and taking into consideration the KM strategy put forth by PAHO, the team reached consensus on Dominica’s goals (Table 2).

**Table 2.** The four goals of a knowledge management strategy to advance the national health agenda in Dominica, 2015

Goal 1	Promote formulation, execution, and evaluation of public policies, standards, and guidelines for the development and sharing of health information and knowledge based on qualified open data.
Goal 2	Strengthen national public health initiatives by effective collaboration and establishment of environments that facilitate creation, access, and circulation of health information and knowledge.
Goal 3	Promote and facilitate partnerships and strategic relationship networks to strengthen activities in the field of knowledge management for advancing the national health agenda.
Goal 4	Promote strategies and programs with regard to information and knowledge management on health that are effective to obtain the individual, social, and policy changes necessary for achievement and maintenance of health.

## CONCLUSIONS

The Internet, social networks, and ICT tools are increasing the amount of structured and unstructured content available online and elsewhere, creating unprecedented possibilities for improving public health actions and informing and transforming societies and governments. More than ever before, people, researchers, and academic and governmental institutions are gaining open, broader, and faster access to data for decision making, innovation, and research. In August 2014, the United Nations Secretary-General Ban Ki-moon asked an Independent Expert Advisory Group to make concrete recommendations for including the data revolution as an essential component of sustainable development (6).

In Dominica, the KM situation analysis described an overall status of the understanding and implementation of information and knowledge management projects, products, and practices. Information about different aspects of knowledge management activities was collected to provide an overall picture to the Minister of Health and the Environment and to his colleagues. The analysis identified what critical knowledge is needed to support overall organizational goals and individual and team activities.

The main conclusion from the analysis was a consensus that a KM strategy will accelerate efficiency and productivity at the MoHE, ultimately improving patient outcomes and quality of care. It was also stated that a KM strategy will contribute to Dominica’s national health agenda by enabling the MoHE to develop its core competencies, as well as to determine the best human and other resources required to achieve the country’s public health goals. A KM strategy will also help monitor progress toward meeting national goals, thereby allowing necessary adjustments to be made continually. Lastly, the team concluded that the implementation of a knowledge management strategy will strengthen the capacity of Dominica’s human resources for health, as well as add innovation, efficiency, and effectiveness to the administrative, technical, and managerial processes of the MoHE.

 Recommendations from the team include strengthening of human resources skills and capacity; identifying the most important resources overall for achieving the national health agenda goals; monitoring of progress toward these goals; bringing efficiency and effectiveness to the delivery of health care services; strengthening management capacity within health services; managing information to facilitate the collection and analysis of appropriate and reliable data for informed decision-making; and providing timely, appropriate, and accurate information to health workers, partners, and to the general population.

## Acknowledgements.

The authors would like to acknowledge the staff of the Ministry of Health and Environment of Dominica and of the Pan American Health Organization for their commitment to developing and implementing the Knowledge Management Strategy for advancing Dominica’s national health agenda.

## Disclaimer.

Authors hold sole responsibility for the views expressed in the manuscript, which may not necessarily reflect the opinion or policy of the *RPSP/ PAJPH *and/or PAHO.
